# A multimodal sleep intervention improves sleep quality and architecture in chronic insomnia: a prospective exploratory study

**DOI:** 10.3389/fmed.2026.1877949

**Published:** 2026-08-18

**Authors:** Yingjie Che, Ranwei Yao, Dan Chen, Xiulin Wu

**Affiliations:** Department of Anesthesiology and Perioperative Medicine, Shihezi People’s Hospital, Shihezi, Xinjiang Uygur Autonomous Region, China

**Keywords:** chronic insomnia, cognitive behavioral therapy for insomnia, dexmedetomidine, propofol, stellate ganglion block

## Abstract

**Background:**

Chronic insomnia is increasingly recognized as a disorder of persistent hyperarousal, and current single-modality treatments often provide limited or unsustained benefits. This study aimed to explore the feasibility, safety, and preliminary signal of benefit of a multimodal sleep intervention combining stellate ganglion block (SGB), cognitive behavioral therapy for insomnia (CBT-I), and anesthetic-assisted sleep induction.

**Methods:**

In this prospective exploratory pilot study, 30 patients with refractory chronic insomnia who showed inadequate response to conventional therapies were enrolled. The intervention consisted of SGB, CBT-I, and propofol- and dexmedetomidine-assisted sleep induction. Subjective sleep quality was assessed using the Pittsburgh Sleep Quality Index (PSQI) at baseline and at 7 days, 1 month, 3 months, and 6 months after treatment. Objective sleep parameters were evaluated using a wearable sleep monitoring device. Repeated measures were analyzed using generalized estimating equations.

**Results:**

PSQI total and component scores were significantly reduced at all follow-up time points compared with baseline (all *p* < 0.05). The mean reduction in PSQI total score was approximately 8 points. Notably, the proportions of deep sleep and REM sleep increased significantly despite minimal changes in total sleep time (both *p* < 0.05). No serious adverse events were observed during the study period.

**Conclusion:**

This multimodal sleep intervention showed preliminary signals of improvements in subjective sleep quality and selected objective sleep architecture parameters, suggesting feasibility and tolerability in patients with refractory chronic insomnia and appeared to be generally well tolerated. Given the exploratory single-arm design and limited sample size, randomized controlled studies are required to further evaluate efficacy, durability, and the relative contribution of individual treatment components.

**Clinical trial number:**

https://www.chictr.org.cn, ChiCTR2400085256.

## 1 Introduction

Insomnia, as one of the most prevalent sleep disorders, is a common phenomenon in daily life, and its prevalence has shown a significant upward trend over recent years. Approximately one-third of the global population experiences insomnia (1). In the United States, 10–15% of individuals suffer from symptoms of insomnia, with 50% of these cases representing chronic insomnia. A large-scale survey conducted in China revealed that 45.4% of the population experienced varying degrees of sleep disorders within the preceding month, and 25% meeting the diagnostic criteria for insomnia (2, 3). Notably, the proportion of insomnia among individuals aged over 65 years has been reported to be as high as 50% (4). Chronic sleep deprivation exerts severe impact on the work and daily life of individuals, making sleep disorders a global issue that cannot be ignored. Furthermore, insomnia is a high-risk factor for multiple systemic diseases, including hypertension, stroke, diabetes, cognitive impairment, and depression.

Conventional treatment modalities primarily include pharmacotherapy and cognitive behavioral therapy for insomnia (CBT-I) (5). Accumulating evidence from clinical trials supports CBT-I as the first-line treatment for chronic insomnia (6–10). Although its short-term efficacy is comparable to that of medication, CBT-I demonstrates superior long-term outcomes (11). However, its clinical practice is limited by factors such as procedural complexity and high cost. Commonly prescribed hypnotic drugs include benzodiazepines, non-benzodiazepine benzodiazepine receptor agonists, dual orexin receptor antagonists, melatonin receptor agonists, melatonin, and sedative antidepressants. Long-term use of these medications may lead to tolerance, resulting in diminished therapeutic efficacy (1). In addition, pharmacologically induced sleep differs from physiological sleep. While these agents can reduce sleep latency, decrease nocturnal awakenings, and prolong total sleep time, they predominantly increase stage N2 sleep (light non-rapid eye movement sleep) at the expense of stage N3 sleep (slow-wave sleep essential for restorative processes) and rapid eye movement (REM) sleep. This alteration in sleep architecture may negatively affect subjective sleep quality despite improvements in total sleep duration (12).

Over recent years, numerous studies have confirmed that ultrasound-guided stellate ganglion block (U-SGB) exerts significant efficacy for the improvement of sleep quality in patients with insomnia (13). U-SGB reduces excitability of the sympathetic nervous system, thereby effectively regulating the balance between circadian rhythm and homeostasis, improving endogenous sleep-related mechanisms, and ultimately enhancing the quality of sleep (14). While exerting therapeutic effects on diseases associated with the innervated region, SGB can exert direct or indirect positive impacts on the systemic autonomic nervous, immune, and endocrine systems (15).

Anesthetic drugs used for sleep induction, such as dexmedetomidine and propofol, have also demonstrated unique efficacy for the treatment of insomnia. Dexmedetomidine, a highly selective *α*₂-adrenoceptor agonist, is primarily characterized by its sedative, analgesic, anxiolytic, and hypnotic effects. The sleep state that is induced by dexmedetomidine closely resembles that of natural sleep (16). As an adrenoceptor agonist, dexmedetomidine modulates a patient’s wakefulness state via the locus coeruleus-norepinephrine pathway, influences other arousal pathways, activates the hypnotic system, reduces hyperarousal, corrects the “sleep–wake” pathway, and improves the quality of sleep (17–20). Propofol is a rapid-onset, short-acting intravenous hypnotic agent that is widely used for the induction and maintenance of general anesthesia, sedation in mechanically ventilated adults, and procedural sedation (21). Previous studies showed that propofol could improve the latency of sleep onset, sleep maintenance, sleep duration, and overall quality of sleep. Furthermore, propofol has been shown to exert protective effects against various central nervous system disorders (22, 23). The use of anesthetic drugs to induce sleep and treat insomnia is not only safe and effective but also improves the onset, maintenance, duration, and overall quality of sleep. Another advantage of this form of therapy is that propofol treatment may help to enhance the responsiveness of patients to hypnotics such as zopiclone or zolpidem, potentially by increasing the pharmacological sensitivity to these drugs (24). However, evidence regarding this approach remains limited, particularly with respect to its medium- and long-term efficacy.

Therefore, the present study aimed to evaluate the short- and medium-term feasibility and preliminary signals of benefit and safety of a treatment regimen combining SGB and CBT-I with propofol and dexmedetomidine-induced sleep in patients with chronic insomnia.

## 2 Methods

### 2.1 General information

**Figure 1:**
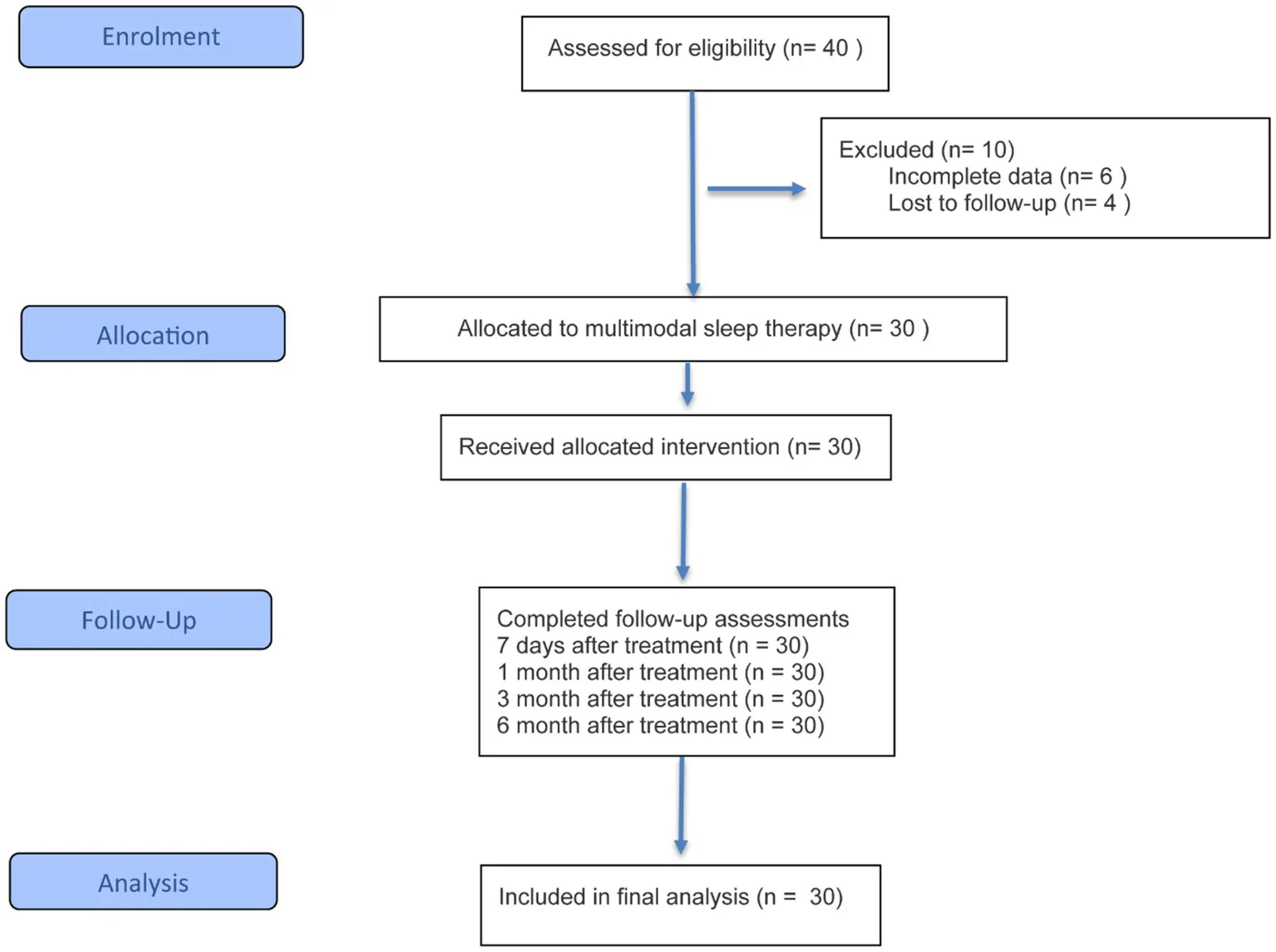
Flow diagram of the prospective single-arm pilot study.

This study was approved by the Ethics Committee of the Shihezi people’s Hospital (Approval No. [2023] 07), and registered with the Chinese Clinical Trial Registry (Registration No. ChiCTR2400085256, https://www.chictr.org.cn). Written informed consent was obtained from all participants. A total of 30 patients diagnosed with chronic insomnia at the Shihezi People’s Hospital, Department of Clinical Psychology between January and December 2025 were enrolled (Figure 1). Eligible participants were adults aged 18–70 years, with a body mass index (BMI) of 18–35 kg/m^2^, classified as American Society of Anesthesiologists (ASA) physical status I–II, and without identifiable sleep-related underlying diseases (Table 1).

**Table 1:** Baseline demographic and clinical characteristics of study participants (*n* = 30).

Characteristic	Value
Demographic characteristics	
Age, mean ± SD (range) (years)	44.7 ± 11.9 (21–67)
Sex, *n* (%)	
Male	7 (23.3%)
Female	23 (76.7%)
Body mass index (BMI), mean ± SD (kg/m^2^)	23.48 ± 3.62
Education level, *n* (%)	
High school or below	7 (23.3%)
Bachelor’s degree	18 (60.0%)
Master’s degree or above	5 (16.7%)
Employment status, *n* (%)	
Full-time employed	20 (66.7%)
Part-time employed	6 (20.0%)
Retired/unemployed	4 (13.3%)
Core insomnia clinical characteristics	
Duration of insomnia, mean ± SD (range) (years)	3.5 ± 2.2 (0.5–11)
Insomnia Severity Index (ISI) score, mean ± SD	18.2 ± 4.5
Prior sleep medication use, *n* (%)	
No prior medication	10 (33.3%)
Benzodiazepines	10 (33.3%)
Z-drugs (zolpidem/eszopiclone)	7 (23.3%)
Antidepressants with hypnotic effects	3 (10.0%)
Duration of prior medication use, mean ± SD (years)	1.7 ± 1.0
Prior CBT-I attempt history, *n* (%)	
No prior attempt	0 (0.0%)
1–2 prior attempts	6 (20.0%)
≥3 prior attempts	24 (80.0%)
Psychological comorbidity assessments	
Self-rating anxiety scale (SAS) score, mean ± SD	55.8 ± 8.9
Self-rating depression scale (SDS) score, mean ± SD	54.3 ± 9.4
Subjective sleep outcome measures	
Pittsburgh sleep quality index (PSQI) total score, median (IQR)	16.50 (14.75, 18.00)
Self-reported time in bed, mean ± SD	556.30 ± 111.40
Objective wearable sleep monitoring baseline measures	
Total sleep time, mean ± SD	258.00 ± 132.10
Sleep efficiency, mean ± SD (min)	47.2 ± 23.5%
Sleep latency, mean ± SD (min)	172.3 ± 131.2
Wake after sleep onset (WASO), mean ± SD (min)	22.93 ± 15.42
Deep sleep time, mean ± SD (min)	107.17 ± 38.42
Deep sleep/total sleep time, mean ± SD	0.27 ± 0.11
REM sleep time, mean ± SD (min)	30.50 ± 45.81
REM sleep proportion, mean ± SD	7.68 ± 11.52%
Somatic comorbidities	
Hypertension	4 (13.3%)
Diabetes mellitus	2 (6.7%)
Chronic pain	3 (10.0%)
No comorbid somatic conditions	21 (70.0%)

Normally distributed continuous variables are presented as mean ± standard deviation (SD); non-normally distributed continuous variables are presented as median (interquartile range, IQR). Categorical variables are presented as number (percentage, %). CBT-I: Cognitive Behavioral Therapy for Insomnia; ISI: Insomnia Severity Index; PSQI: Pittsburgh Sleep Quality Index; SAS: Self-rating Anxiety Scale; SDS: Self-rating Depression Scale; WASO: Wake after sleep onset; REM: Rapid Eye Movement.

The inclusion criteria were as follows: (1) patients meeting the diagnostic criteria for chronic insomnia according the Chinese Guidelines for the Diagnosis and Treatment of Adult Insomnia (2023 Edition) (25); (2) prior treatment in accordance with the aforementioned guidelines, involving at least two classes of therapeutic drugs for more than 6 months with poor or no clinical response; and (3) a Pittsburgh Sleep Quality Index (PSQI) score ≥10 at admission, consistent with a diagnosis of chronic insomnia. All participants were enrolled voluntarily and provided written informed consent.

The exclusion criteria were as follows: (1) history of sleep apnea syndrome, epilepsy, known alcoholism, or drug dependence; (2) pregnancy or lactation; (3) presence of psychiatric disorders, those taking antipsychotic medications, or severe impairments in vision, hearing, or verbal communication; and (4) concomitant hepatic or renal dysfunction, cardiopulmonary insufficiency, cerebrovascular disease, or hematological system disorders.

### 2.2 Study design

#### 2.2.1 Treatment protocol

Treatment was divided into a treatment phase and a consolidation phase, as described below.

##### 2.2.1.1 Treatment phase

1. Pre-treatment preparation: (i) Patient assessment: Patients completed the Insomnia History Collection Form, Insomnia Severity Index (ISI), PSQI, Somatic Symptom Self-rating Scale, Epworth Sleepiness Scale (ESS), Self-rating Anxiety Scale (SAS), and Self-rating Depression Scale (SDS); (ii) Informed consent: Written informed consent was obtained for U-SGB and sleep disorder treatment; and (iii) Baseline sleep monitoring: Patients were equipped with a wearable sleep monitoring device to record baseline sleep parameters.
2. Day 1: (i) Bilateral SGB was performed (left side first, followed by the right side 30 min later); (ii) A 30-min, one-on-one session of CBT-I focused on sleep education was conducted. Following treatment, patients returned home wearing the monitoring device to enable objective sleep recording and completed sleep diaries.
3. Day 2: (i) Left-sided SGB was administered; (ii) A 30-min one-on-one CBT-I session was conducted; (iii) At 21:00, intravenous infusion of propofol and dexmedetomidine was initiated to induce sleep over a 2-h period. After discontinuation, patients were allowed to awaken naturally and were accompanied home by family members to continue sleep; sleep diaries were recorded.
4. Day 3: (i) Right-sided SGB was performed; (ii) A 30-min one-on-one CBT-I session was conducted. Patients returned home for natural sleep and recorded sleep diaries.
5. Day 4: (i) Left-sided SGB was administered; (ii) A 30-min one-on-one CBT-I session was conducted; (iii) At 21:00, intravenous infusion of propofol and dexmedetomidine was administered for sleep induction over a 2-h period. After discontinuation, patients were allowed to awaken naturally and were accompanied home by family members; sleep diaries were recorded.
6. Day 5: (i) Right-sided SGB was performed; (ii) A 30-min one-on-one CBT-I session was conducted. Patients returned home for natural sleep and recorded sleep diaries.
7. Day 6: (i) Left-sided SGB was administered; (ii) A 30-min one-on-one CBT-I session was conducted. Patients returned home for natural sleep and recorded sleep diaries.
8. Day 7: (i) Bilateral SGB was performed (left side first, followed by the right side 30 min later); (ii) A 30-min one-on-one CBT-I session was conducted. After treatment, patients returned home wearing the monitoring device for objective sleep recording and completion sleep diaries.

##### 2.2.1.2 Consolidation phase

During the following 3 weeks, unilateral SGB was performed twice weekly (Tuesdays and Fridays), with alternating left and right sides.

#### 2.2.2 Technique and monitoring methods

##### 2.2.2.1 SGB technique

The SGB procedure was performed by trained anesthesiologists under ultrasound guidance using a color Doppler ultrasound diagnostic system (Mindray M9; Shenzhen Mindray Bio-Medical Electronics Co., Ltd., Shenzhen, China). Patients were placed in the supine position with the neck slightly rotated to the contralateral side. Following standard disinfection of the neck skin, a high-frequency linear array probe was selected. After applying coupling gel, the probe was covered with a sterile glove and placed at the level of the sixth cervical vertebra (C6) to identify the transverse process of C6 and the C6 nerve root. The probe was positioned transversely at the level of the cricoid cartilage. Gentle pressure was applied to the probe to center the carotid artery on the screen. The point of needle insertion was identified on the skin at the lateral edge of the probe. Under ultrasound monitoring, a 27-gauge needle was advanced using an in-plane approach toward the longus colli muscle beneath the prevertebral fascia. After negative aspiration for blood, 5 mL of 1% lidocaine hydrochloride injection (Batch No. D32507092, Hubei Jin Yao Pharmaceutical Co., Ltd.) was injected. Under ultrasound, the spread of the solution was observed as a crescent- or fusiform-shaped anechoic area on the surface of the longus colli muscle and posterior to the carotid sheath, potentially extending in cranial and caudal directions (C6–C7 levels).

##### 2.2.2.2 Sleep induction technique

All patients fasted for 8 h and were instructed to refrain from fluids for 2 h prior to treatment, as per routine protocol. At 21:00, patients entered the monitoring unit for sleep induction. Upon entering the room, routine monitoring was initiated by a professional attending anesthesiologist, including bi-spectral index (BIS), blood pressure, heart rate, and pulse oximetry. A professional anesthesia nurse established peripheral venous access. An intravenous bolus of propofol (of 0.5–1.5 mg/kg; Batch No. 2508162, Sichuan Guorui Pharmaceutical Co., Ltd.) was concurrently administered with a 10-min infusion of dexmedetomidine (0.5–1.0 μg/kg; Batch No. 19081031, Yangtze River Pharmaceutical Group Co., Ltd.). Subsequently, continuous infusion of propofol (2–4 mg/kg/h) and dexmedetomidine (0.2–0.7 μg/kg/h) was maintained, with dosages titrated to achieve a BIS value between 50 and 70. The infusion was continued for 2 h, with dexmedetomidine discontinued 30 min prior to completion. Following cessation of the infusion, patients were allowed to awaken naturally. After confirmation of normal vital signs, monitoring was discontinued, and patients were discharged home, where they were permitted to resume oral intake and sleep naturally. Low-flow oxygen was administered via face mask throughout the procedure. Patients were closely observed during the administration of medication. The procedure was immediately terminated if systolic blood pressure fell below 100 mmHg or heart rate dropped below 50 beats per minute and did not respond to appropriate interventions, such as intravenous fluid bolus or atropine administration.

##### 2.2.2.3 CBT-I technique

CBT-I was delivered by a qualified clinical psychologist in 30-min, one-on-one sessions. The intervention aimed to establish healthy sleep-related cognitions and behavioral patterns. Individualized sleep–wake schedules were developed based on the specific sleep–wake characteristics of each patient, aiming to reduce wake time after bedtime and increase sleep drive. Building upon traditional CBT-I, the approach incorporated the management of patient anxiety and depression, provided appropriate psychological counseling, and enhanced patient confidence in recovering from insomnia. All participating clinical psychologists received standardized, systematic professional training focused specifically on CBT-I protocols before conducting the intervention, including standardized operation procedures, patient communication skills and adjustment strategies for insomnia-related comorbidities.

Moreover, treatment fidelity was strictly assessed throughout the whole research period. We adopted two approaches to guarantee treatment consistency: (1) All CBT-I sessions were recorded with patients’ written informed consent; (2) Senior supervisors with rich experience in CBT-I randomly reviewed session recordings on a regular basis using a standardized fidelity checklist. Any deviations from the standard CBT-I protocols were timely corrected via one-on-one supervision.

##### 2.2.2.4 Wearable sleep monitoring device

A Xiaomi Smart Band 8 Pro (Xiaomi Technology Co., Ltd., Beijing, China) was used for objective monitoring. Baseline sleep parameters collected prior to treatment included total sleep time (TST), duration of deep sleep, light sleep, and the REM sleep (26–28). The monitoring bracelets are uniformly provided by the hospital. The current procedure requires patients to visit the hospital for bracelet fitting and return the device the next day without receiving any other clinical interventions during the interval. This protocol necessitates two separate hospital visits for each patient. Restricted by poor patient compliance, most patients show low willingness to cooperate, resulting in low clinical implementability of this procedure. For future research, we will guide patients to acquire monitoring bracelets on their own to facilitate data collection.

### 2.3 Outcome measures

Outcome measures included the following: (1) Objective sleep parameters were assessed using a Xiaomi Smart Band 8 Pro before treatment and at 7 days after treatment only, due to practical limitations of long-term wearable device data collection. These data are considered exploratory due to the consumer-grade nature of the device and the lack of comprehensive clinical validation for sleep stage classification; (2) Subjective sleep quality was assessed using the PSQI at baseline and at 7 days, 1 month, 3 months, and 6 months after treatment. Follow-up assessments at these time points were conducted via telephone interviews or an online questionnaire platform (Questionnaire Star); (3) Adverse events associated with anesthesia, such as nausea and dizziness, were observed and recorded.

### 2.4 Evaluation criteria

Clinical efficacy was defined as follows: Patients were considered cured if sleep returned to normal (PSQI score ≤ 5). Treatment was considered effective if the PSQI total score was reduced by ≥ 8 points from baseline. Treatment was considered ineffective if there was no improvement in sleep quality. The overall response rate was calculated as: (number of cured cases + number of effective cases) / total number of cases × 100%. Sleep quality was assessed using the PSQI, which evaluates seven dimensions: subjective sleep quality, sleep duration, sleep efficiency, sleep disturbances, sleep latency, use of hypnotic medication, and daytime dysfunction. Each dimension was scored from 0 to 3. A total score ≥ 6 indicated impaired sleep quality, with higher scores reflecting poorer sleep quality.

### 2.5 Statistical analysis

This study was designed as a prospective, single-arm superiority trial. The primary efficacy endpoint was the change in total PSQI score from baseline to the end of treatment. Sample size estimation was performed using G*Power software (Heinrich-Heine-Universität Düsseldorf, version 3.1). A recent study (29), reported that standardized CBT-I for chronic insomnia reduced the mean PSQI score by 5.0 points (standard deviation = 4.5). In the present study, we hypothesized that multi-modal sleep therapy could increase this improvement to 8.0 points. With a one-sided significance level (*α*) of 0.05 and a statistical power (1-*β*) of 80%, the calculated effect size (Cohen’s *d*) was 0.67. Accordingly, a minimum of 17 evaluable cases was required. Assuming a dropout or missing data rate of approximately 25%, the planned sample size was 23 participants.

A total of 40 patients were initially enrolled to account for a higher anticipated dropout rate. Of these, six patients were excluded due to incomplete core data resulting from poor treatment adherence, and another four patients declined post-treatment follow-up for personal reasons, leading to missing longitudinal follow-up data. Accordingly, these 10 patients were eliminated from the study cohort, 30 patients were included in the final analysis. Statistical analysis was performed using SPSS (version 23.0). As the data did not meet the assumption of normality, generalized estimating equation (GEE) was employed for repeated-measures analysis. *p* value <0.05 was considered statistically significant. To address the skewed distribution and extreme outliers of REM sleep metrics, log transformation was applied to REM duration and REM proportion prior to generalized estimating equation modeling. For these non-normal variables, GEEs with a gamma distribution family and log link function were utilized, while normally distributed sleep architecture parameters were analyzed via Gaussian GEE with an identity link. No participants were excluded solely due to outlying values; complete case analysis was only implemented for patients with fully missing wearable recordings. Descriptive statistics are presented as untransformed raw values (mean ± SD) for clinical interpretability.

## 3 Results

### 3.1 Baseline characteristics

The summary analysis indicates that the study cohort comprised predominantly middle-aged females (mean age 44.7 years, 76.7% female) with a mean insomnia duration of 3.5 years. Most patients had moderate-to-severe insomnia (median PSQI 16.50, mean ISI 18.2), and 80% had undergone ≥ 3 prior CBT-I attempts, confirming the refractory nature of their condition. Psychological assessments revealed clinically elevated anxiety (SAS mean 55.8) and depression (SDS mean 54.3) scores. Baseline sleep architecture was characterized by markedly reduced REM sleep proportion (mean 7.68%) (Table 1).

### 3.2 Changes in total PSQI score and component scores before and after treatment

Compared with baseline, repeated measures analysis revealed that PSQI scores were significantly lower after treatment (*p* < 0.001). The median total score decreased from 16.50 before treatment to 9.50 after treatment, and remained at relatively low levels of 9.50, 9.00, and 8.50 at the 1-, 3-, and 6-month follow-ups, respectively. With regards to individual PSQI components, significant improvements were observed in all components except for Factor 6 (use of sleep medication), which did not show a statistically significant change (*p* = 0.108) (Table 2).

**Table 2:** Changes in Pittsburgh Sleep Quality Index (PSQI) total and component scores from baseline to follow-up time points.

Indicator	Before treatment	After treatment				Wald χ^2^ value	*P*-value
		7 Days	1 Month	3 Months	6 Months		
F1: Sleep quality	3.00 (2.00, 3.00)	1.00 (1.00, 2.00)ᵃ	1.00 (0.75, 2.00)ᵃ	1.00 (1.00, 2.00)ᵃ	1.00 (1.00, 2.00)ᵃ	103.73	< 0.001
F2: Sleep onset latency	3.00 (3.00, 3.00)	2.00 (1.00, 3.00)ᵃ	1.50 (1.00, 3.00)ᵃ	2.00 (1.00, 3.00)ᵃ	2.00 (1.00, 3.00)ᵃ	45.44	<0.001
F3: Sleep duration	3.00 (1.75, 3.00)	1.50 (1.00, 3.00)ᵃ	2.00 (1.00, 2.00)ᵃ	1.00 (1.00, 2.00)ᵃ	1.00 (0.00, 3.00)ᵃ	31.68	<0.001
F4: Sleep efficiency	3.00 (2.75, 3.00)	1.00 (0.00, 3.00)ᵃ	1.50 (0.00, 3.00)ᵃ	2.00 (1.00, 3.00)ᵃ	1.00 (0.00, 3.00)ᵃ	35.60	<0.001
F5: Sleep disturbances	2.00 (1.00, 2.00)	1.00 (1.00, 1.00)ᵃ	1.00 (1.00, 1.25)ᵃ	1.00 (1.00, 1.00)ᵃ	1.00 (1.00, 1.00)ᵃ	27.58	<0.001
F6: Use of sleeping medication	3.00 (0.00, 3.00)	1.50 (0.00, 3.00)	0.50 (0.00, 3.00)	0.00 (0.00, 3.00)	1.00 (0.00, 3.00)	7.59	0.108
F7: Daytime dysfunction	2.00 (1.75, 3.00)	1.00 (0.75, 2.00)ᵃ	1.00 (0.75, 2.00)ᵃ	1.00 (0.00, 2.00)ᵃ	1.00 (0.00, 1.00)ᵃ	51.52	<0.001
Total score	16.50 (14.75, 18.00)	9.50 (7.75, 12.00)ᵃ	9.50 (6.50, 12.00)ᵃ	9.00 (6.75, 13.25)ᵃ	8.50 (5.75, 13.00)ᵃ	115.59	<0.001

The omnibus Wald χ^2^ global tests (overall inter-timepoint differences) do not require Bonferroni adjustment, as these single global tests do not involve repeated pairwise comparisons. The *post-hoc* pairwise timepoint comparisons marked with superscript ‘a’ were analyzed without Bonferroni correction. ᵃ*P* < 0.05 vs. baseline.

After one complete course of treatment, all outcome indicators showed a gradual upward trend from baseline to the 6-month follow-up. The effective rate increased from 0.00% at baseline to 26.60% immediately after treatment, remained stable at 26.60% at 1 month, and further increased to 36.67 and 40.00% at 3 and 6 months, respectively. The cure rate increased 0.00% at baseline to 6.66% immediately after treatment, reached 13.33% at 1 month, remained stable at 13.33% at 3 months, and further increased to 26.67% at 6 months. The sleep efficiency rate improved from 30.00% at baseline to 80.00% immediately after treatment, decreased to 66.67% at 1 month, and subsequently increased to 73.33 and 86.67% at 3 and 6 months, respectively. The proportion of patients reducing sleep medication increased progressively from 0.00% at baseline to 40.00, 46.00, 50.00, and 53.33% at the immediate, 1-, 3-, and 6-month time points, respectively. Similarly, the overall effective rate increased from 0.00% at baseline to 46.67% immediately after treatment, further increased to 50.00% at 1 month and 53.33% at 3 months, and remained stable at 53.33% at 6 months (Table 3).

**Table 3:** Changes in clinical efficacy and medication use over time in patients with chronic insomnia (*n* = 30).

Evaluation indicator	Before treatment (%)	After treatment (%)			
		Immediate	1 Month	3 Months	6 Months
Effective rate	0.00	26.60	26.60	36.67	40.00
Cure rate	0.00	6.66	13.33	13.33	26.67
Sleep efficiency rate	30.00	80.00	66.67	73.33	86.67
Medication reduction rate	0.00	40.00	46.00	50.00	53.33
Overall effective rate	0.00	46.67	50.00	53.33	53.33

Effective rate: proportion of patients with a ≥ 8-point reduction in PSQI total score from baseline. Cure rate: proportion of patients with a post-treatment PSQI score ≤ 5. Sleep efficiency rate: proportion of patients achieving PSQI Factor 4 score ≤ 2. Medication reduction rate: proportion of patients with PSQI Factor 6 score of 0. Overall effective rate: proportion of patients classified as cured or effective.

### 3.3 Changes in smart band recorded sleep parameters before and after treatment

Wearable sleep monitoring data further showed (Table 4) that total sleep time and light sleep time remained comparable before and after intervention (both *p* > 0.05). Deep sleep duration increased by 19.00 min from baseline, corresponding to an approximate relative growth of 17.7%, though this difference failed to reach statistical significance (*p* = 0.067). The deep sleep ratio significantly rose by 3.0 percentage points, equivalent to a relative increment of 11.1% (*p* = 0.010). Wake after sleep onset was markedly shortened after treatment (*p* = 0.002). In addition, the duration of REM sleep showed statistically meaningful improvements relative to baseline (*p* = 0.012). After data correction, the median wearable-recorded REM proportion rose from 0.00 (0.00, 0.17) at baseline to 0.15 (0.00, 0.23) after intervention (*p* = 0.011). However, it is important to note that the baseline zero median REM proportion is physiologically implausible and likely reflects the Xiaomi Smart Band’s known limitation in detecting REM sleep during periods of frequent nocturnal micro-arousals in insomnia patients. After intervention, reduced nighttime restlessness may have lowered motion artifacts and improved the band’s REM detection sensitivity, which partially explains the apparent rise in recorded REM value rather than pure physiological restoration of sleep architecture.

**Table 4:** Changes in sleep architecture measured by wearable sleep monitoring device before and after treatment (*n* = 30).

Indicator	Before treatment	Post-treatment	*P*-value
Total sleep time (min)	397.40 ± 72.35	416.70 ± 65.82	0.498
Deep sleep time (min)	107.17 ± 38.42	126.17 ± 31.56	0.067
Light sleep time (min)	236.80 ± 131.60	232.77 ± 68.24	0.873
Wake after sleep onset (min)	22.93 ± 15.42	4.83 ± 3.21	0.002*
Deep sleep/total sleep time	0.27 ± 0.11	0.30 ± 0.05	0.010*
REM sleep time (min)	30.50 ± 45.81	52.93 ± 44.22	0.012*
REM sleep proportion (%)	0.00 (0.00, 0.17)	0.15 (0.00, 0.23)	0.011*

Descriptive data are presented as mean ± standard deviation (SD). All between-time-point *p*-values were calculated via generalized estimating equations (GEEs). An exchangeable working correlation structure, Gaussian distribution family with identity link function were adopted for normally distributed indicators (total sleep time, deep sleep time, light sleep time, deep sleep proportion); for non-normally distributed indicators (REM sleep time, REM sleep proportion, wake after sleep onset), GEEs with Gamma distribution family and log link function were used. Missing data were handled via complete case analysis, and 10 participants with incomplete longitudinal data were excluded before analysis. Within each indicator, only two time points (baseline vs. post-treatment) were compared. No multiple comparison adjustment was conducted for time-point contrasts. *P < 0.05 indicates a statistically significant difference.

### 3.4 Safety analysis

All procedures were performed under continuous monitoring by trained anesthesiologists and anesthesia nurses. No complications related to U-SGB, such as brachial plexus block or hoarseness, were observed. During the sleep induction phase, no patients experienced decreases in blood pressure, heart rate, or oxygen saturation below predefined safety thresholds. Following the procedure, no adverse events, such as nausea or vomiting, were reported.

## 4 Discussion

Chronic insomnia, characterized by its long-term and chronic course, can substantially impair cognitive function. With the advancement of medical technology, anesthetic sleep induction techniques have gradually been applied in the clinical management of chronic insomnia. Although the quality of sleep improves with propofol anesthesia alone for sleep induction, the depth of sleep and cognitive function do not improve, and euphoria may occur during the emergence period. Over recent years, dexmedetomidine has been widely used for perioperative and ICU sedation, improving postoperative sleep, with its safety and reliability extensively confirmed (30). Furthermore, dexmedetomidine has been reported to increase the concentration of brain-derived neurotrophic factor, which may be associated with neuroprotective effects in other clinical contexts (31, 32). The combined use of propofol and dexmedetomidine may mitigate adverse effects associated with high-dose monotherapy, such as bradycardia and hypotension, while enabling better control of sedation depth. This combination was part of the multimodal intervention, and its specific contribution to the overall outcomes cannot be isolated in the present study.

In the present study, we found that following multi-modal sleep therapy, the total PSQI score decreased significantly compared with baseline. This trend suggests that the intervention provides both rapid symptom relief and sustained therapeutic benefits over time. For patients receiving long-term pharmacological treatment, abrupt discontinuation of sleep medications should be avoided due to the risk of rebound insomnia and severe psychiatric symptoms (25). In this study, no significant changes were observed in the sleep medications following treatment. However, the proportion of patients reducing medication use increased progressively during follow-up, exceeding 50% at 6 months, indicating a gradual reduction in medication dependence over time. Notably, the cure rate and the proportion of patients achieving sleep efficiency targets were generally higher than the overall response rate and medication reduction rate, suggesting that even patients who did not meet the predefined PSQI reduction threshold still experienced clinical meaningful improvements in sleep quality or remission. Approximately half of the patients experienced rapid symptom relief shortly after treatment, with sustained efficacy observed during long-term follow-up.

The PSQI is one of the most widely used subjective assessment scales in clinical practice, demonstrating good reliability and reasonable concordance with polysomnography for evaluating sleep quality and screening for sleep disorders (33). Although polysomnography remains the gold standard for assessing sleep architecture, its clinical application is limited by cost and practicality. Actigraphy-based assessments have shown acceptable agreement with polysomnography in multiple studies (34–36). In this study, sleep was objectively monitored using a wearable device. The Xiaomi Smart Band 8 Pro employs photoplethysmography to track heart rate and oxygen saturation, combined with motion sensors (accelerometer and gyroscope) to capture activity and positional changes. Integrated algorithms analyze heart rate variability, oxygen saturation, and movement characteristics to estimate sleep stages, including deep sleep, light sleep, REM sleep, and wakefulness. Daytime dysfunction resulting from insomnia primarily includes fatigue, low mood or irritability, physical discomfort, cognitive impairment, and anxiety (37). In the present study, although total sleep time did not significantly increase, daytime dysfunction improved significantly, indicating an overall enhancement in subjective sleep experience. Exploratory data from the wearable device suggested potential alterations in sleep architecture, particularly a significant increase in REM sleep time following the multimodal intervention, though these findings require validation with polysomnography. REM sleep is closely associated with memory consolidation, particularly the conversion of short-term memory to long-term memory, emotional regulation, learning new skills, and brain development. However, the mechanism underlying the selective increase in REM sleep without significant changes in total sleep time or deep sleep time remains unclear and warrants further investigation.

Notably, we observed a substantial discrepancy between the device-measured wake after sleep onset (WASO; 22.93 ± 15.42 min at baseline) and the subjective sleep parameters. Given the baseline subjective sleep efficiency of 47.2% and a mean self-reported time in bed of 556 min, the total wake time during the in-bed period is estimated at approximately 260 min. Even after accounting for the prolonged sleep onset latency of 172.3 min, approximately 90 min of wakefulness remain unaccounted for. This discrepancy strongly suggests that the consumer-grade wearable device underestimates WASO, a known limitation of photoplethysmography (PPG)-based wearables. These devices rely on heart rate variability and accelerometry to classify sleep stages, and they frequently misclassify quiet wakefulness (e.g., lying still while awake) as light sleep, particularly in individuals with insomnia who may exhibit motionless periods of wakefulness (38). This limitation further underscores the exploratory nature of all wearable-derived sleep architecture findings in this study, and future studies should employ validated polysomnography to confirm these preliminary observations. Furthermore, the baseline zero median REM proportion cannot represent the true physiological sleep structure of insomnia patients. Polysomnography gold-standard studies consistently confirm that patients with primary chronic insomnia always retain detectable REM fragments, with complete overnight REM absence only observed in fatal familial insomnia, pontine lesions, or acute high-dose sedative exposure—populations excluded in this trial. The zero REM readout is a known limitation of consumer-grade Xiaomi wearables: these PPG + accelerometer devices fail to identify subtle REM signals amid frequent nocturnal limb micro-arousals in insomnia patients, leading to severe REM undercounting at baseline (26). Therefore, the apparent increase in REM proportion after intervention may partially reflect improved device detection sensitivity due to reduced movement artifacts, rather than exclusively representing a true physiological restoration of sleep architecture.

Multi-modal sleep therapy involves the combination of multiple treatment modalities (39). Zhang et al. (39) reported a case of refractory insomnia treated with a multi-modal approach combining dexmedetomidine, propofol, and ketamine with transcranial magnetic stimulation, SGB, and CBT-I. Although intranasal dexmedetomidine has been reported to improve sleep latency and duration, its use in insomnia remains off-label and is not currently recommended (Level B evidence) (40). Complementary feasibility and retrospective studies further support anesthetic-behavioral multimodal strategies beyond this single case report. An et al. (41) verified the feasibility of patient-controlled intravenous dexmedetomidine combined with standardized behavioral sleep training for intractable insomnia; this simplified single-sedative regimen achieved favorable sleep restoration with fewer adverse effects, providing prospective pilot evidence to supplement Zhang’s case-based observations. For patients with insomnia complicated by anxiety and depression, Li et al. (42) proposed another multimodal paradigm integrating ultrasound-guided SGB and myofascial release with sleep behavioral interventions. SGB mitigates autonomic hyperarousal, a key barrier limiting standalone CBT-I efficacy, and serves as a non-intravenous alternative for patient intolerant to intravenous sedation.

The 2023 European insomnia guideline (43) provides high-level consensus for these combinatorial therapies. It recognizes CBT-I as first-line management yet notes poor responses in refractory, mood-comorbid patients, and clarifies that anesthetic/neuromodulatory techniques function as adjuncts to potentiate behavioral therapy rather than monotherapies. Nevertheless, all existing relevant evidence consists of case reports, single-arm feasibility trials and retrospective cohorts, failing to reach Level A evidence per guideline standards. Future randomized controlled trials are warranted to directly compare intravenous dexmedetomidine-based multimodal therapy, SGB-combined interventions and isolated CBT-I, to clarify their long-term efficacy and safety profiles.

While both CBT-I and pharmacotherapy are effective for the short-term insomnia management; complete remission is rarely achieved with a single modality (44). In this study, we a multi-modal approach combining SGB, CBT-I anesthetic-assisted sleep induction significantly improved PSQI scores and increased the proportion of REM sleep. This multimodal integrative strategy appears to offer a feasible and potentially effective treatment option for patients with chronic refractory insomnia who respond poorly to conventional therapies, although the individual contribution of each component requires further investigation. However, the sleep architecture findings are based on a non-validated consumer device and should be interpreted with caution.

### 4.1 Significance and limitations

By comparing symptomatic improvements before and after treatment for the same patients, we observed that a multi-modal insomnia treatment strategy centered on anesthetic techniques was associated with rapid symptom alleviation in this preliminary feasibility study, increase the proportion of deep sleep in the short-term, and enhance patient confidence, with sustained efficacy was observed over a 3–6 month follow-up period. Compared with polysomnography, wearable devices were used to obtain objective sleep data. Such devices enable continuous monitoring of sleep characteristics, including duration, efficiency, quality, regularity, and physiological parameters related to autonomic function (45). Although objective monitoring suggested alterations in sleep architecture, particularly an increase in REM sleep time, these findings should be interpreted with caution due to the limitations of the wearable device. The observed changes may reflect algorithm-based estimations rather than true physiological alterations. Therefore, we consider these results preliminary and hypothesis-generating, rather than definitive evidence of improved sleep architecture. They provide a practical and scalable alternative to traditional assessments, although with certain limitations.

This study has several limitations. First, the sample size was relatively small, and the single-arm design limits the generalizability of the findings. Second, inter-individual variability in medication reduction was observed, warranting further investigation. Third, the follow-up period was limited to 6 months, and repeat treatment was not administered to patients with relapse. Future studies should include larger sample sizes, control groups, and longer follow-up periods. Randomized controlled trials are needed to confirm these preliminary signals and to establish efficacy, as the current single-arm design does not permit causal attribution to any individual treatment component or exclusion of regression to the mean or placebo effects, and to elucidate the underlying biological mechanisms, as well as to evaluate the efficacy and safety of repeated treatment courses in patients with suboptimal initial response. Furthermore, future studies with larger sample sizes should incorporate intention-to-treat analyses to address potential attrition bias. We acknowledge that the use of a consumer-grade wearable device (Xiaomi Smart Band 8 Pro) based on photoplethysmography and accelerometry for sleep staging is a significant limitation. Its accuracy for detecting specific sleep stages, particularly REM sleep, is not validated against polysomnography, the gold standard, and may be subject to considerable variability and potential algorithm errors. Furthermore, given the single-arm design, it is important to consider potential interaction effects among the three treatment components. For example, SGB may reduce sympathetic hyperarousal, thereby facilitating the effects of CBT-I on sleep-related anxiety and cognitive restructuring. Anesthetic-assisted sleep induction may provide immediate relief from sleep debt, potentially enhancing patients’ engagement and responsiveness to CBT-I. Conversely, CBT-I may reinforce the behavioral and cognitive benefits initiated by the other modalities. However, the relative magnitude and direction of these interactions remain speculative and require future studies with factorial designs to disentangle the contribution of each component.

## Data Availability

The original contributions presented in the study are included in the article/supplementary material, further inquiries can be directed to the corresponding author/s.
